# Editorial: Highlights in Hypertension: 2021

**DOI:** 10.3389/fcvm.2022.926949

**Published:** 2022-06-15

**Authors:** Guido Iaccarino

**Affiliations:** Center for Research on Hypertension and Related Conditions, Federico II University of Naples, Naples, Italy

**Keywords:** hypertension, endothelium, meta-analysis, public health, clinical trial

## Introduction

Hypertension remains the most relevant cardiovascular risk around the world, and many aspects of the physiopathology and clinical management of this condition are still to be evaluated. Frontiers in Cardiovascular Medicine—Hypertension metrics of the last years have positioned the journal in the high ranks of specialized journals, with wide-open perspectives of consolidating their position in the near future. This collection aims to celebrate the seven top cited and accessed articles published by the journal in 2021 (Table 1).

**Table 1:** Impact of the papers of the Highlights in Hypertension 2021 collection as of 4 May 2022.

**Title**	**Downloads**	**Views**	**Citations**
Acute Effects of Cocoa Flavanols on Blood Pressure and Peripheral Vascular Reactivity in Type 2 Diabetes Mellitus and Essential Hypertension: A Protocol for an Acute, Randomized, Double-Blinded, Placebo-Controlled Cross-Over Trial	818	1,602	1
Antidepressant Drugs Effects on Blood Pressure	1,471	19,293	1
Association Between Triglyceride-Glucose Index and Hypertension: A Meta-Analysis	644	1,244	6
Bacterial-Induced Blood Pressure Reduction: Mechanisms for the Treatment of Hypertension via the Gut	312	2,564	2
Barriers, Enablers and Strategies for the Treatment and Control of Hypertension in Nepal: A Systematic Review			
Downregulation of Cullin 3 Ligase Signaling Pathways Contributes to Hypertension in Preeclampsia	711	1,282	2
L-Arginine Improves Cognitive Impairment in Hypertensive Frail Older Adults	21	306	0

A total of 7 out of over 70 papers published from 1 January to 31 December 2021 have been selected, that have been most read and cited, with the perspective to influence research in the near future. Interestingly, this collections is composed of a well-calibrated mixture of basic, clinical, and meta-analysis research, together with two reviews on hot topics for hypertension and public health perspectives of the burden of this silent killer. The fact stands that Frontiers in Cardiovascular Disease—Hypertension has the ability to be interdisciplinary, putting together all scientific stakeholders from lab technicians to clinicians to policy advisors. In this editorial we present these papers, organized by subheadings.

## Endothelium as a Mechanism of Disease

From the analysis of the top accessed papers, we also learn that the investigation of the mechanisms of the disease is still a major drive for hypertension research, and in particular the endothelium remains the focus of many investigators around the world.

As anticipated above, the endothelium is still a trending topic of hypertension research. Interestingly, the relevance of endothelial dysfunction is investigated in never-explored clinical implications of hypertension. The group of Santulli and others introduces the important concept that the observed impairment of cognitive function in hypertension is indeed measurable with classical neurophysiological assessments (like the MoCA test), and can be reversed by treatment (Mone et al.). The authors even provide a pathophysiological explanation, by suggesting that endothelial dysfunction can be responsible for the cognitive impairment, and that arginine can indeed ameliorate both. Many issues remain open in this regard that need to be explored in larger studies with age-matched normotensives, also considering the comorbidities and cardiovascular risk evaluation. Also, mild cognitive function is hard to assess using MoCA, and probably cognitive tests that are easier to administer like the questionnaire for mild cognitive impairment (1) could facilitate the diffusion of the assessment of cognitive function in the larger population of hypertensive patients. This will finally allow researchers to include the assessment of brain function in the evaluation of hypertension-related target organ damage (2, 3).

Again, a defect in endothelial function is also pivotal for another threatening condition, preeclampsia. In this case, the Zhang laboratory at the Shanghai Jiaotong University in China showed that impaired vasodilation, but also reabsorption of volume by the kidney in preeclampsia can be induced by the downregulation of the signaling of specific ligases (CURL3) (Zhang et al.). This set of ligases is therefore key to the balance of vasoconstriction and vasodilation and sodium reabsorption. Whether other ligases are also involved in such mechanisms and in other hypertensive states remains the open question.

## Clinical Trial Design

Also with the endothelium in mind, Patrick Calders for the University of Gent and his group present a clinical trial that could shed light on the use of cocoa flavonoids in the management of hypertensive patients (Tanghe et al.). They are inspired by the increasing relevance of food supplements and functional foods in the management of acute and chronic conditions. In their study, they will investigate whether cocoa flavanols have an acute impact on blood pressure and vascular reactivity in patients with type 2 diabetes with and without arterial hypertension. Almost 800 mg of flavonoids in a capsule will be taken by patients and the acute effects will be assessed on vasodilation using near-infrared spectroscopy to assess forearm muscle vasoreactivity. Again, the endothelium represents the target of this strategy for modulating peripheral resistances.

## Hot Topics in Hypertension

In 2021, two highly cited reviews focused on hot topics in hypertension: microbiota and behavioral disorders. The paper by Cookson from the University of Alberta, Canada, illustrates the mechanisms through which several metabolites that are produced in the gut by the living microbiota can interfere with distant organs, such as the gut, brain, kidneys, liver, heart, vasculature, and host immunity. These substances modulate the regulation of blood pressure homeostasis. The interesting emerging implication is that the use of selected microbiota strains could produce or reduce the production of appropriate metabolites to elicit a net anti-hypertensive effect. Indeed, the review proposes the gut microbiota as a possible therapeutic target in hypertension.

The group of Aderville Cabassi in Parma, Italy reviewed the available evidence regarding the effects of several antidepressant drugs on blood pressure regulation, with a focus on the hypo- and hypertensive responses of these drugs (Calvi et al.). The issue is of growing interest, especially in the post COVID-19 season, with many patients suffering from depression due to the restriction imposed in the last years. Interestingly, many options are available for hypertensive patients to take care of this condition without having a significant impact on their blood pressure, nevertheless, researchers should use caution, and the administration of antidepressants requires a pathophysiological reasoning.

## Biomarkers

Going back to the physiopathology of hypertension, the investigation of easily accessible biomarkers of insulin sensitivity represents the aim of a continuous quest. In 2021, this paper investigated the predictive role of the novel triglyceride/glucose ratio (the TyG index) for hypertension, by performing a meta-analysis of observational studies around the world (Wang et al.). Results showed that compared with those with the lowest category of the TyG index, subjects with the highest category of the TyG index were associated with higher odds of hypertension. This index is therefore proposed as a possible predictor of hypertension among the general population, thus favoring a public health preventive intervention.

## Public Health Approaches to Tackle Hypertension

Again, a public health approach for the evaluation of the management of high blood pressure in a small (28 M people living) south-Asian country, Nepal (Dhungana et al.) shows the barrier and the constrains for the management of this condition. The health system in Nepal faces daunting challenges such as unequal distribution of health care services, poor infrastructures, inadequate supply of essential drugs, poorly regulated private providers, inadequate budget allocation for health, and poor retention of human resources in rural areas (4). In this context, the management of hypertension as a public health problem is exemplificative of such a situation: most hypertensive individuals are not aware of their condition (awareness = 40.0%); a half of those aware are not treated (treatment = 20.2%), and in half of those on treatment, the blood pressure was not controlled (control = 10.5%). The systematic review of the literature by the Australian group led by Maximilian De Courtain identifies gaps and barriers in the management of this public health problem and suggests specific interventions to tackle this problem in this corner of South Asia.

## Conclusions

The year 2021 represents a very important year for the growth of the journal, which has been led by specialty editor Professor Michel Burnier, to whom is addressed my personal gratitude for mentoring the associate editors and shaping the directions of the journal. With the growing visibility and number of submissions we are facing in this quarter, we can expect a further development of the journal that aims to increase its reputation among researchers of the field of hypertension. Frontiers in Cardiovascular Medicine—Hypertension aims to consolidate the reputation among researchers and, at the same time, become the arena where researchers around the world, working on the several realms of hypertension, wish to showcase their results.

## Author Contributions

GI reviewed the literature and wrote the manuscript.

## Funding

GI was supported by a grant from the Italian Ministry of Research PRIN (2017HTKLRF).

## Conflict of Interest

The author declares that the research was conducted in the absence of any commercial or financial relationships that could be construed as a potential conflict of interest.

## Publisher's Note

All claims expressed in this article are solely those of the authors and do not necessarily represent those of their affiliated organizations, or those of the publisher, the editors and the reviewers. Any product that may be evaluated in this article, or claim that may be made by its manufacturer, is not guaranteed or endorsed by the publisher.
